# The need for a telemedicine strategy for Botswana? A scoping review and situational assessment

**DOI:** 10.1186/s12913-020-05653-0

**Published:** 2020-08-26

**Authors:** B. Ncube, M. Mars, R. E. Scott

**Affiliations:** 1 Department of TeleHealth, College of Health Sciences, University of KwaZulu-Natal, Durban, South Africa; 2 Dynamics Research & Development Institute, Gaborone, Botswana; 3 Department of Community Health Sciences, University of Calgary, Calgary, Alberta Canada

**Keywords:** Telemedicine, Telehealth, eHealth, Strategy, Botswana

## Abstract

**Background:**

Health, healthcare, and healthcare system problems within the developing world are well recognised. eHealth, the use of Information and Communications Technologies (ICT) for health, is frequently suggested as one means by which to ameliorate such problems. However, to identify and implement the most appropriate ehealth solutions requires development of a thoughtful and broadly evidence-informed strategy. Most published strategies focus on health informatics solutions, neglecting the potential for other aspects of ehealth (telehealth, telemedicine, elearning, and ecommerce). This study examined the setting in Botswana to determine the need for a telemedicine-specific strategy.

**Methods:**

A situational assessment of ehealth activities in Botswana was performed through a scoping review of the scientific and grey literature using specified search terms to July 2018; an interview with an official from the major mhealth stakeholder; and benchtop review of policies and other relevant Government documents including the country’s current draft eHealth Strategy.

**Results:**

Thirty-nine papers were reviewed. Various ehealth technologies have been applied within Botswana. These include Skype for educational activities, instant messaging (WhatsApp for telepathology; SMS for transmission of laboratory test results, patient appointment reminders, and invoicing and bill payment), and robotics for dermatopathology. In addition health informatics technologies have been used for surveillance, monitoring, and access to information by healthcare workers. The number of distinct health information systems has been reduced from 37 to 12, and 9 discrete EMRs remain active within the public health institutions. Many infrastructural issues were identified. A critical assessment of the current draft ehealth strategy document for Botswana showed limitations. Many telemedicine services have been introduced over the years (addressing cervical cancer screening, teledermatology, teleradiology, oral medicine and eye screening), but only one project was confirmed to be active and being scaled up with the intervention of the Government.

**Conclusions:**

Botswana’s draft ‘ehealth’ strategy will not, in and of itself, nurture innovative growth in the application of telemedicine initiatives, which currently are fragmented and stalled. This lack of focus is preventing telemedicine’s recognised potential from being leveraged. A specific Telemedicine Strategy, aligned with and supportive of the pre-existing ehealth strategy, would provide the necessary focus, stimulus, and guidance.

## Background

Health and healthcare problems in developing countries abound, and include disease burden, poor doctor to patient ratios, lack of access to health specialists, and shortages of drugs and medicines. Although ICT4D (ICT for Development) is advancing and presenting opportunities to address such health problems, most developing countries find it challenging to use ICT for health activities, termed eHealth.

Despite multiple definitions and misuse of terms, and although some people use the terms ehealth, telehealth and telemedicine interchangeably, it is globally accepted that these terms are distinct [1]. The World Health Organization (WHO) defines ehealth as ‘the use of information and communication technologies (ICT) for health’ [2], and it can be considered an umbrella term. Telehealth, a component of eHealth, refers to the provision of both clinical and non-clinical healthcare services, education, and training delivered through ICT. It has been defined as the use of ICTs to exchange health information and provide healthcare services across geographic, time, social, cultural, and political barriers, and the breadth of technology, application, and role of telehealth is large [1]. In turn, telemedicine is a component of telehealth and refers to clinical healthcare services delivered through ICT [3]. The primary focus of this paper is on telemedicine in Botswana.

Botswana is a large landlocked country with an estimated population for 2018 of 2,249,104, 45% of whom live in rural areas [4], a population density of ~ 4 people per km^2^, a low doctor to patient ratio (3.8/10,000) [2] and a high HIV/AIDS prevalence (22.8% for ages 15–49) [5]. There are few medical specialists with, for example, only one dermatologist in the public health sector, with most specialists residing in urban areas. In alignment with the Abuja Declaration 2001 [6] the Government allocates 15% of GDP (USD$756 Million, 2018/2019 budget) to health. The national health delivery system, in line with Botswana’s Ministry of Health and Wellness ‘Integrated Health Services Plan’ (IHSP), consists of six levels: 3 tertiary (referral) hospitals (Francistown, Gaborone, Lobatse), 18 general hospitals, 17 primary hospitals, 318 clinics (104 with beds), 347 health posts, and 973 mobile stops, with services delivered by 830 doctors, and 7427 nurses [7]. These health facilities are operated by the Ministry of Health and Wellness (MOH), private institutions, faith based organisations and mining companies.

Although Botswana has a high active SIM card subscriber rate of 157% [8], with major cities, towns and villages connected to the Internet, Internet use by world standards is still low at 39.4% [9]. Despite the ubiquity of mobile Internet globally, ICT utilisation for healthcare in Botswana is limited. Consequently, the potential of ICT4D for ehealth and telemedicine is yet to be realised.

The WHO proposes ehealth as a facilitator of health, and sees an ehealth strategy as a prerequisite and an enabler to ehealth implementation. Most countries have discrete and uncoordinated ehealth activities. In Botswana three components of ehealth exist, health information systems (EMRs), telehealth (mhealth), and elearning (use of mobile phones to access PubMed databases and retrieve medical information). The health informatics implementations are incomplete and only a few of the available modules have been installed. mHealth telemedicine initiatives are limited and funder dependent and most have stopped. In 2015, with the assistance of WHO, Botswana developed a draft ehealth strategy, premised on the WHO/ITU eHealth Strategy Toolkit [10]. The draft strategy aimed to have telehealth services, mhealth, and a standard telehealth infrastructure in place by 2016, however this has not occurred because the strategy has yet to be approved.

In the absence of an approved ehealth strategy and noting the need for rural access to specialist and other healthcare, telemedicine may address some of the current health system’s shortcomings and deficiencies in healthcare provision and delivery. This could be facilitated by a telemedicine specific strategy. This would require a review of the status of ehealth activities within Botswana, an understanding of what might need to be addressed, analysis of the draft ehealth strategy with respect to telemedicine, and identification of potential barriers to the development of a telemedicine strategy. The aim of this study was to investigate the need, if any, for a specific telemedicine strategy for Botswana.

## Methods

Three distinct methods were used: a scoping review of the literature, an interview with an official from the major mhealth stakeholder, and benchtop review of policies and other relevant Government documents. The scoping review was conducted to provide a broad review of ehealth activities in Botswana, and used an adaptation of published guidelines [11]. Online electronic databases (PubMed and Science Direct) were searched for publications up to July 2018. Search terms were developed for both databases and included; telemedicine, ehealth strategy, health records, cell phones, organisation, administration, developing countries, and Botswana. Search strings used for PubMed are shown in Table 1.

**Table 1:** Search strings used for searching PubMed

String	Search terms
1	((“telemedicine”[MeSH Terms] OR “telemedicine”[All Fields]) AND (“organisation”[All Fields] OR “organization and administration”[Subheading] OR (“organization”[All Fields] AND “administration”[All Fields]) OR “organization and administration”[All Fields] OR “organization”[All Fields] OR “organizations”[MeSH Terms] OR “organizations”[All Fields]) AND (“Botswana”[MeSH Terms] OR “Botswana”[All Fields]))
2	((“telemedicine”[MeSH Terms] OR “telemedicine”[All Fields]) AND Strategy [All Fields] AND (“organisation”[All Fields] OR “organization and administration”[Subheading] OR (“organization”[All Fields] AND “administration”[All Fields]) OR “organization and administration”[All Fields] OR “organization”[All Fields] OR “organizations”[MeSH Terms] OR “organizations”[All Fields] OR “organization and administration”[MeSH Terms] OR (“organization”[All Fields] AND “administration”[All Fields]) OR “administration”[All Fields]) AND (“Botswana”[MeSH Terms] OR “Botswana”[All Fields]))
3	((“telemedicine”[MeSH Terms] OR “telemedicine”[All Fields] OR “telehealth”[All Fields]) AND (“Botswana”[MeSH Terms] OR “Botswana”[All Fields]))
4	((“medical records”[MeSH Terms] OR “medical records”[All Fields] OR (“medical”[All Fields] AND “records”[All Fields])) AND Systems [All Fields] AND (“Botswana”[MeSH Terms] OR “Botswana”[All Fields]))
5	((“telemedicine”[MeSH Terms] OR “telemedicine”[All Fields]) OR e-health [All Fields] OR “ehealth”[All Fields]) AND (“cell phone”[MeSH Terms] OR (“cell”[All Fields] AND “phone”[All Fields]) OR “cell phone”[All Fields] OR “cellphone”[All Fields]) AND (“Botswana”[MeSH Terms] OR “Botswana”[All Fields]))

Google Scholar was searched using the terms Botswana, telemedicine, EMR, and ehealth strategy. Grey literature was searched using Google (first 100 hits), and 10 official government websites were also searched. Titles and abstracts of potential resources were first reviewed independently by all authors for relevance using the following inclusion criteria; the resource addressed one or more of: ehealth or telemedicine implementation within Botswana; ICT infrastructure within Botswana; ehealth or telemedicine strategy in developing countries; development or application of ehealth strategy; or ehealth strategy for a healthcare facility, health region, or country; plus the papers were in English. Full papers of identified resources were further assessed against the inclusion criteria and reviewed in the same way. Hand searching supplemented the database searches. All final selections were based upon consensus. Data were abstracted for study type, technology, health information technology (HIT), training, strategy, and clinical (telemedicine) services, and synthesised into these key themes.

As eighteen papers were generated by the Botswana-UPenn partnership (between the University of Pennsylvania, the Government, and the University of Botswana), the official was interviewed to establish the status of their projects. Policies and other government documents relevant to telemedicine in Botswana were identified and reviewed as part of the situational assessment. In particular the draft Botswanan eHealth Strategy 2016–2018 document was critically reviewed to understand its strengths and weaknesses in relation to telemedicine, and to gather insight regarding the status of telemedicine projects.

## Results

**Fig. 1:**
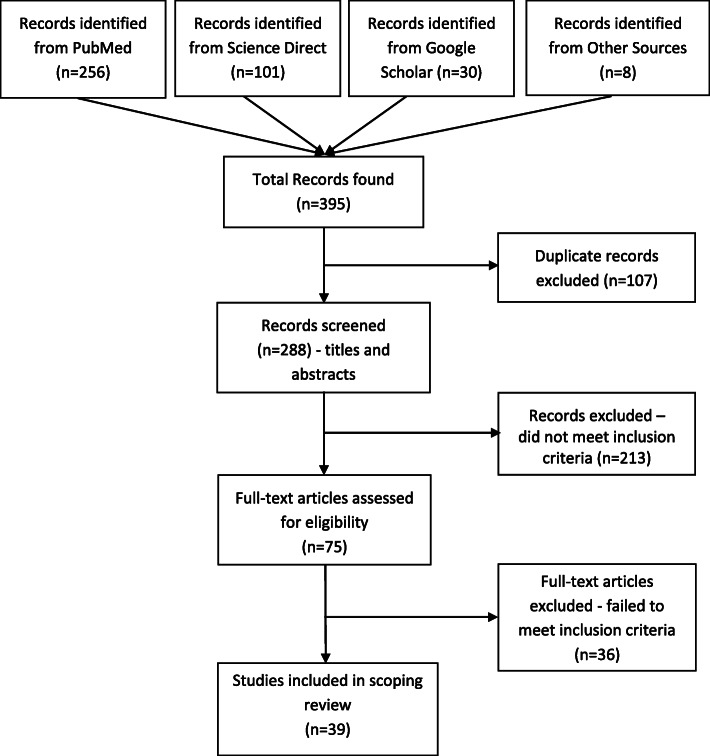
Flow diagram of the search strategy and search results

Thirty-nine resources were included in the review (Fig. 1). There were 27 journal articles, two book chapters, one letter to the editor, one special report to PAHO, one systematic review protocol, one systematic review, one poster abstract and five institutional documents. The results show that substantive ehealth initiatives exist in Botswana, which are first summarised below, before focussing on telemedicine activity. For the literature summary, retrieved papers were categorised into five foci: technology, health information technology (HIT), training, strategy, and clinical services (telemedicine).

### Literature findings

The technology focus included applications involving Skype, SMS, and robotics. Skype has been used to share patient information and deliver educational services between Botswana and Canada [12]. Short Message Service (SMS) applications have included transmission of CD4 test results from the laboratory to the clinic [13], patient appointment reminders [14], and invoicing and bill payment [15]. Telerobotics for dermatopathology was reported which was replaced with telepathology using WhatsApp [16].

HIT activities within Botswana have covered surveillance or monitoring for TB [17–19], and access to information by healthcare workers [20]. In addition Coppock et al. [21] reported on a pharmacy mobile application used to track antiviral medication refill data. Nine discrete EMRs have been identified and remain active within the public health institutions [22–24]. Between 2012 and 2015 the government reduced the number of HIT solutions from 37 to 12 [24] and now focusses on the Integrated Patient Management System (IPMS), District Health Information System 2 (DHIS2), Central Stores Drug Management, and Patient Information Management System (PIMS) [10].

Several applications have been applied to training or education. Okrainec et al. [25] used Skype for simulation to teach Laparoscopic Surgery in Botswana, whilst Armstrong et al. [26] described SMS for clinical practice guidelines. Witt et al. [27] examined the use of tablets in mlearning, and the University of Botswana School of Medicine (UBSOM)/UPenn partnership developed a mobile learning programme for training medical students [28]. Finally, Oladokun [29] examined the information needs and information seeking behaviour of the populace, and described the responsibility of the government (and others) and associated policy in establishing an information rich setting for the country.

No study described the process of ehealth strategy development within the developing world. However, strategy related to ICT and ehealth overall has been developed in Botswana. In 2004, the Government developed an ICT strategy to guide the development of the country’s ICT infrastructure branded Maitlamo [30]. The policy assisted the country in developing an almost nationwide ICT infrastructure, including a fibre backbone network (BOFINET), making viable other ICT related initiatives such as egovernment and ehealth. A first draft of an ehealth strategy was embedded in the ICT Policy of 2004, but only as an appendix [31]. In 2012 the Botswana Parliament released its ICT Master Plan in which the intent to pursue an ehealth Botswana programme was identified [32]. This document also recognised and differentiated telemedicine (described as “curative services provided through telecommunication technologies, including telephone, videoconferencing and Internet”) and telehealth (described as “wider healthcare advice provided through telecommunications”). In 2015, Botswana released version 4 of a specific ehealth strategy [10]. This draft document provided a detailed single framework to help all the key stakeholders in the health sector to implement the required ehealth initiatives.

Clinical services (telemedicine) have also taken place within Botswana. Most studies (18) have been performed by a single research group, the BUP Partnership [13, 18, 20, 21, 26–28, 33–43]. The available evidence suggests this partnership is no longer functional. These studies have largely been on mobile telemedicine (mhealth), concentrated around the capital city Gaborone, and report on the same four mhealth pilot projects. The projects addressed cervical cancer screening, teledermatology, teleradiology and oral medicine telemedicine services. Only the PEEK project, the Portable Eye Examination Kit, was confirmed to still be active.

Telemedicine services have addressed cervical cancer screening, teledermatology, teleradiology, oral medicine and eye screening. Quinley et al. [41] studied the diagnostic agreement between remote diagnosis by a gynaecologist using photographs of the cervix and in-person visual inspection with acetic acid by midwives. The images for remote evaluation were taken using a mobile phone camera and transmitted through multimedia messaging service (MMS).

Azfar et al. [33] examined the reliability and accuracy of outcomes derived from the store and forward images. Subsequently, Azfar et al. [34] investigated mobile dermatology for taking and transmitting images of skin lesions in HIV positive patients. Schwartz et al. [42] compared chest X-rays with digital photos taken from a mobile phone, opening the possibility of access to the three radiologists practising in Botswana’s public health system, all located in Francistown and Gaborone. Mobile phones have been used by local health practitioners to collect and upload patient information for oral medicine purposes, sending it to a remote server for second opinion [43]. PEEK has been adopted in several countries and uses a smartphone app and a low-cost adapter for retinal imaging to screen for core vision problems [44]. The BUP Partnership, in collaboration with the Standard Chartered Bank Botswana, MOH, Ministry of Education and Botswana Optometrists Association, commissioned PEEK Botswana, a national eye screening and treatment management project for school children [45]. In addition, Joubert et al. [46] explored the use of screening to address hypertension in an urban population from Gaborone, and proposed a telemedicine-supported model of care as a potential solution.

### Interview

The BUP official was interviewed solely to confirm the status of their telemedicine projects. Most were non-functional. Only one project remains active because donor funding has ceased, and the Government could not take on the projects once the pilot phases were completed. Only the PEEK project is active and is being scaled up with the intervention of the Government.

### Botswana ehealth strategy review

The 2015 document ‘Botswana eHealth Strategy v4’ [10] was critically reviewed to understand the strengths and weakness of the Strategy, in particular in relation to telemedicine. The document adopted the WHO definition for ehealth. However, there were omissions, oversights, and biases identified that impair the utility of the current draft strategy. For example, although the terms telehealth, telemedicine, and mhealth are mentioned there are no clear definitions provided of these and other key terms. Further, the general tone of the document is very ‘informatics’ focussed, with the term ‘telemedicine’ used just 5 times but ‘health information’ 17 times.

Connected to these issues, there is no clear description of the depth and breadth of interventions possible through non-informatics ehealth approaches. The draft eHealth Strategy also failed to emphasise the over-riding need for interventions to address specific and identifiable health needs. Although some health status data for Botswana is presented, and improvement in Millenium Development Goals (MDGs) has been emphasised, there is little consideration of the broad identification of health issues. Of further note was the lack of any consideration of identifying non-eHealth solutions to identified health needs. In addition, although mention was made of ‘high-priority eHealth services and applications’ there was no description of how priority was determined, nor if or how any priority was given to identified health needs before consideration of eHealth solutions.

### Barriers

The reviewed literature identified infrastructure issues as the main barrier to implementation of telemedicine in developing countries, including Botswana. According to Oluoch et al. [47] issues included “unstable electrical power, loss of Internet connectivity, and access to mobile phones.” Low levels of computer literacy, lack of end user training, bandwidth cost, lack of clinical and technical expertise, poor user acceptance, increased workload of medical practitioners and poor network coverage were documented as additional barriers to mobile telemedicine [22, 48]. Other barriers were weak organisational capacities, lack of technological training, lack of evidence based decision making, lack of financing, lack of strong political leadership (free of corruption), negative attitudes towards use of ICT, lack of technological awareness, resistance to new processes, poor data quality, and a lack of development of data standards [48].

## Discussion

This study used a scoping review of the literature, an interview with a Botswana/UPenn official, and review of government policies and documents to provide a current situational assessment of the state of ehealth, and particularly telemedicine, within Botswana. The study findings from the 39 literature sources and interview have demonstrated telemedicine’s potential to provide access equivalent to face-to-face clinical services, and to reduce the inconvenience and costs of travel by patients to clinics. Many barriers were identified from the literature also. In addition, a critical assessment of the current draft ehealth strategy document for Botswana showed limitations. The combined findings highlight the absence of scalable and sustainable telemedicine activity, which supports the need for development and implementation of a telemedicine-specific strategy for Botswana.

An effective strategy must identify and plan to overcome barriers. Although the application of telemedicine is promising, notable infrastructural constraints remain such as poor connectivity, bandwidth costs, increased workload of medical practitioners, unreliable network coverage, and lack of clinical and technical expertise, training, stable electrical power, and user acceptance [22, 47]. Some resources highlighted more subtle issues such as a culture of not using ‘technology’, lack of financing, lack of strong political leadership, and lack of business modelling as other important issues [48, 49].

Clinical activities were reported in teleradiology, teledermatology, telepathology, cervical cancer screening, and oral medicine using mobile applications. Although there were eighteen papers reporting mhealth telemedicine initiatives supported by UPenn these have not been sustained beyond the pilot phase. To date, only one telemedicine project (PEEK) is being scaled-up by the MOH. Therefore, although debatable, while Botswana might need telemedicine more than any other ehealth component, current evidence now shows little government activity in this direction. Ironically, this significant gap in telemedicine also presents an opportunity. An interesting observation is the spontaneous emergence of WhatsApp activities that are self-initiated, self-sponsored and driven by medical professionals [50] with similar activity noted in Botswana [16]. However, critical issues such as data protection, privacy and confidentiality, ethical, regulation and legal compliance are beginning to emerge. Unless these issues are addressed, they too remain potential barriers to this form of telemedicine.

Perhaps the most significant barrier is the absence of a focussed strategy for telemedicine. The Botswana government developed and implemented an ICT Policy (Maitlamo), culminating in a sound ICT environment, covering most cities, towns and villages. In 2007 an eHealth strategy was produced as an appendix to the ICT Policy, with little mention of telemedicine. Because of its presentation as only an appendix, the strategy document did not achieve the expected objectives. A separate draft ehealth strategy was written in 2015 with ambitious objectives expected to be achieved by 2016. These were never achieved. In 2018 the first draft was superseded by the second ehealth strategy draft. As long as the current document remains a draft, it will lack the commitment it deserves.

It is likely the weaknesses identified have led to a lack of focussed attention given to structured growth, development and application of telemedicine. For example, the draft eHealth Strategy has a general ‘informatics’ tone, and it lacks depth and breadth of description of potential telemedicine applications. There is also a lack of association of potential telemedicine applications with specific and identifiable health needs. Although mentioned in the draft strategy the Global Burden of Disease study, which provides risk factors that drive the most death and disability for Botswana [51], could be further leveraged. This weakness is further compounded by the subsequent lack of examination of possible solutions for the identified (and prioritised) health issues (whether or not they require ehealth intervention) including their feasibility, cost etc.. Collectively, these weaknesses prevent the reader from having a comprehensive understanding of needs and corresponding options available. Addressing these weaknesses would lead to more rational decision-making about appropriate ehealth, and specifically telemedicine, opportunities [52] and have an important impact on the future health and quality of life of Batswana and the cost to the system for their healthcare.

When developing a strategy document, consideration should be given to additional literature resources that provide clear fundamental principles and steps for strategy development [52]. Solutions to specific health issues may require a predominance of one component (health informatics, telehealth, elearning, ecommerce) over others, and it is likely that any sustainable and comprehensive solution will require elements of each [52]. This was recently emphasised by Novillo-Ortiz et al. [53] who noted that the PAHO eHealth Strategy actively promotes the use of telehealth, telemedicine, mhealth, elearning and education for health using ICTs, not only health informatics (HIT; e.g., electronic medical records). Without a clear focus in the form of a specific strategy, it is unlikely that telemedicine will blossom in Botswana.

### Limitations

The study focused on the setting in Botswana, but relevant insight concerning telemedicine strategies may have been gained from looking more broadly. Thus the study did not assess the existence of telemedicine specific strategies from other African countries.

## Conclusions

Given the absence of an approved ehealth strategy, the need for rural access to specialist and other healthcare resources, and existing evidence of the application of telemedicine to address some of the other current health system shortcomings and deficiencies, greater emphasis on telemedicine implementation is appropriate. However, to achieve scaled and sustained application of telemedicine solutions a specific telemedicine strategy is required.

## Data Availability

The study primarily used secondary sources of data identified within the references. Unpublished contemporaneous notes were made of the interview with the BUP official.

## Abbreviations

BOCRA: Botswana Communications Regulatory Authority
BUP: Botswana University of Pennsylvania Partnership
EMR: Electronic Medical Records
GDP: Gross Domestic Product
HIT: Health Information Technology
ICT: Information and Communications Technologies
ICT4D: Information Communication Technologies for Development
IHME: Institute for Health Metrics and Evaluation
MDGs: Millenium Development Goals
PAHO: Pan American Health Organization
PEEK: Portable Eye Examination Kit
PIMS2: Patient Information Management System Version 2
SMS: Short Message Service
TEET: Technology Enabled and Enhanced Training
UPenn: University of Pennsylvania
WHO/ITU: World Health Organization/International Telecommunications Union

## References

[1] Scott RE, Mars M (2015). Telehealth in the developing world: current status and future prospects. Smart Homecare Technol Telehealth.

[2] World Health Organisation. eHealth at WHO. World Health Organisation. 2018. https://www.who.int/ehealth/en/. Accessed 20 June 2019.

[3] Weinstein RS, Krupinski EA, Doarn CR (2018). Clinical examination component of telemedicine, telehealth, mHealth, and connected health medical practices. Med Clin North Am.

[4] The World Bank. Rural Population (% of total population). The World Bank. 2017. https://data.worldbank.org/indicator/sp.rur.totl.zs. Accessed 20 June 2019.

[5] Avert. HIV and AIDS in Botswana Avert. 2018. https://www.avert.org/professionals/hiv-around-world/sub-saharan-africa/botswana. Accessed 17 June 2019.

[6] World Health Organisation. The Abuja Declaration: Ten Years On. https://www.who.int/healthsystems/publications/Abuja10.pdf. Accessed 20 June 2018.

[7] Statistics Botswana. Annual Report 2017-2018. Statistics Botswana 2017. http://www.statsbots.org.bw/sites/default/files/documents/Statistics%20Botswana%20Annual%20Report%202018.pdf. Accessed 20 June 2019.

[8] Botswana Communications Regulatory Authority (BOCRA). BOCRA Annual Report BOCRA. 2018. https://www.bocra.org.bw/sites/default/files/documents/BOCRA_Annual_Report_2018.pdf. Accessed 23 June 2019.

[9] Stats ITU. Percentage of individuals using the internet. ITU. 2018; https://www.itu.int/en/ITU-D/Statistics/Documents/statistics/2018/Individuals_Internet_2000-2017.xls. Accessed 10 Dec 2018.

[10] Ministry of Health, Republic of Botswana. The eHealth Strategy of Botswana-2016-2020. FINAL DRAFT v4 at 30/11/15. Gaborone, Botswana. 2015.

[11] Tricco AC, Lillie E, Zarin W, O'Brien KK, Colquhoun H, Levac D (2018). PRISMA extension for scoping reviews (PRISMA-ScR): checklist and explanation. Ann Intern Med.

[12] Armfield NR, Bradford M, Bradford NK (2015). The clinical use of Skype—for which patients, with which problems and in which settings? A snapshot review of the literature. Int J Med Inform.

[13] Dryden-Peterson S, Bennett K, Hughes MD, Veres A, John O, Pradhananga R (2015). An augmented SMS intervention to improve access to antenatal CD4 testing and ART initiation in HIV-infected pregnant women: a cluster randomized trial. PLoS One.

[14] Michael J, Dhar SI, Mark C, Liang P, Thompson J, Gabaitiri L (2014). Opinions and attitudes of participants in a RCT examining the efficacy of SMS reminders to enhance antiretroviral adherence: a cross sectional survey. J Acquir Immune Defic Syndr Hum Retrovirol.

[15] Botswana Medical Aid Society (BOMAID). Bomaid Annual Report 2012. BOMAID. 2012. https://bomaid.co.bw/wp-content/uploads/2016/07/Bomaid-Annual-Report-2012-1.pdf. Accessed 23 June 2019.

[16] Williams V, Kovarik C (2018). WhatsApp: an innovative tool for dermatology care in limited resource settings. Telemed J E Health.

[17] Alpers L, Chrouser K, Halabi S, Moeti T, Reingold A, Binkin N (2000). Validation of the surveillance system for tuberculosis in Botswana. Int J Tuberc Lung Dis.

[18] Ha YP, Littman-Quinn R, Antwi C, Seropola G, Green RS, Tesfalul MA (2013). A mobile health approach to tuberculosis contact tracing in resource-limited settings. Stud Health Technol Inform.

[19] Vranken R, Coulombier D, Kenyon T, Koosimile B, Mavunga T, Coggin W (2002). Use of a computerized tuberculosis register for automated generation of case finding, sputum conversion, and treatment outcome reports. Int J Tuberc Lung Dis.

[20] Park E, Masupe T, Joseph J, Ho-Foster A, Chavez A, Jammalamadugu S (2016). Information needs of Botswana health care workers and perceptions of wikipedia. Int J Med Inform.

[21] Coppock D, Zambo D, Moyo D, Tanthuma G, Chapman J, Re VL (2017). Development and usability of a smartphone application for tracking antiretroviral medication refill data for human immunodeficiency virus. Methods Inf Med.

[22] Akhlaq A, Sheikh A, Pagliari C (2015). Barriers and facilitators to health information exchange in low- and middle-income country settings: a systematic review protocol. J Innov Health Inform.

[23] Bussmann H, Wester CW, Ndwapi N, Vanderwarker C, Gaolathe T, Tirelo G (2006). Hybrid data capture for monitoring patients on highly active antiretroviral therapy (HAART) in urban Botswana. Bull World Health Organ.

[24] Ndlovu K, Mogotlhwane T, Scott RE, Mars M: E-Health interoperability landscape: Botswana. Botswana. 2016. In: Mmopelwa G, Sebona NM, Prakash J, Anderson GO, editors. *Proceedings of the 6th IASTED African Conference on Environment and Water Resource Management. Africa EWRM 2016–837 Health Informatics, 838 Modelling and Simulation, 839 Power and Energy Systems; 2016 Sep 5–7; Gaborone, Botswana*. Calgary, Canada ACTA Press; 2016. Session-837-010.

[25] Okrainec A, Henao O, Azzie G (2010). Telesimulation: an effective method for teaching the fundamentals of laparoscopic surgery in resource-restricted countries. Surg Endosc.

[26] Armstrong K, Liu F, Seymour A, Mazhani L, Littman-Quinn R, Fontelo P (2012). Evaluation of txt2MEDLINE and development of short messaging service-optimized, clinical practice guidelines in Botswana. Telemed J E Health.

[27] Witt RE, Kebaetse MB, Holmes JH, Littman-Quinn R, Ketshogileng D, Antwi C (2016). The role of tablets in accessing information throughout undergraduate medical education in Botswana. Int J Med Inform.

[28] Chang AY, Ghose S, Littman-Quinn R, Anolik RB, Kyer A, Mazhani L (2012). Use of mobile learning by resident physicians in Botswana. Telemed J E Health.

[29] Oladokun O. Moving towards a ubiquitous service for information access: the information environment in Botswana. In: *Concepts and Advances in Information Knowledge Management.* Edited by Bwalya KJ, Mnjama NM, Sebina PMIIM. Oxford: Chandos Publishing; 2014: 193–211.

[30] Republic of Botswana, Botswana. Ministry of Information and Communication technology: Maitlamo. Botswana's national ICT policy. Legislative framework and change report. Gaborone. December 2004.

[31] Appendix D - e-Health Botswana; Botswana’s National ICT Policy 2004. United Nations Public Administration Network (UNPAN) 2004. http://unpan1.un.org/intradoc/groups/public/documents/cpsi/unpan028383.pdf. Accessed 16 June 2019.

[32] Republic of Botswana: Botswana Parliament ICT Master Plan. August 2012**.** Parliament of Botswana, Gabororone, Botswana: 2012. https://www.uneca.org/sites/default/files/PublicationFiles/botswana-parliament-ict-master-plan.pdf. Accessed 29 June 2019.

[33] Azfar RS, Weinberg JL, Cavric G, Lee-Keltner IA, Bilker WB, Gelfand JM (2011). HIV-positive patients in Botswana state that mobile teledermatology is an acceptable method for receiving dermatology care. J Telemed Telecare.

[34] Azfar RS, Lee RA, Castelo-Soccio L, Greenberg MS, Bilker WB, Gelfand JM (2014). Reliability and validity of mobile teledermatology in human immunodeficiency virus-positive patients in Botswana: a pilot study. JAMA Dermatol.

[35] Chavez A, Littman-Quinn R, Ndlovu K, Kovarik CL (2015). Using TV white space spectrum to practise telemedicine: a promising technology to enhance broadband internet connectivity within healthcare facilities in rural regions of developing countries. J Telemed Telecare.

[36] Fischer MK, Kayembe MK, Scheer AJ, Introcaso CE, Binder SW, Kovarik CL (2011). Establishing telepathology in Africa: lessons from Botswana. J Am Acad Dermatol.

[37] Goldbach H, Chang AY, Kyer A, Ketshogileng D, Taylor L, Chandra A (2014). Evaluation of generic medical information accessed via mobile phones at the point of care in resource-limited settings. J Am Med Inform Assoc.

[38] Littman-Quinn R, Luberti AA, Kovarik C (2013). mHealth to revolutionize information retrieval in low and middle income countries: introduction and proposed solutions using Botswana as reference point. Stud Health Technol Inform.

[39] Littman-Quinn R, Mibenge C, Antwi C, Chandra A, Kovarik CL (2013). Implementation of m-health applications in Botswana: telemedicine and education on mobile devices in a low resource setting. J Telemed Telecare.

[40] Ndlovu K, Littman-Quinn R, Park E, Dikai Z, Kovarik CL (2014). Scaling up a Mobile telemedicine solution in Botswana: keys to sustainability. Front Public Health.

[41] Quinley KE, Gormley RH, Ratcliffe SJ, Shih T, Szep Z, Steiner A (2011). Use of mobile telemedicine for cervical cancer screening. J Telemed Telecare.

[42] Schwartz AB, Siddiqui G, Barbieri JS, Akhtar AL, Kim W, Littman-Quinn R (2014). The accuracy of mobile teleradiology in the evaluation of chest X-rays. J Telemed Telecare.

[43] Tesfalul M, Littman-Quinn R, Antwi C, Ndlovu S, Motsepe D, Phuthego M (2013). Evaluating the impact of a mobile oral telemedicine system on medical management and clinical outcomes of patients with complicated oral lesions in Botswana. Stud Health Technol Inform.

[44] Giardini ME (2015). The portable eye examination kit: Mobile phones can screen for eye disease in low-resource settings. IEEE pulse.

[45] Ndlovu K, Mauco KL, Littman-Quinn R. Telemedicine in Low Resource Settings: A Case for Botswana. In: *Health Information Systems and the Advancement of Medical Practice in Developing Countries.* Edited by Moahi KH, Bwalya KJ, Sebina PM. Hershey: IGI Global; 2017: 129–148.

[46] Joubert J, Nkomazana O, Mompati K, Joubert L, Preux PM, La Croix P (2014). A community survey of cardiovascular risk factors in an urban population in Botswana exploring potential for telemedicine. Eur Res Telemed.

[47] Oluoch T, Santas X, Kwaro D, Were M, Biondich P, Bailey C (2012). The effect of electronic medical record-based clinical decision support on HIV care in resource-constrained settings: a systematic review. Int J Med Inform.

[48] Akhlaq A, McKinstry B, Muhammad KB, Sheikh A (2016). Barriers and facilitators to health information exchange in low-and middle-income country settings: a systematic review. Health Policy Plan.

[49] van Limburg M, van Gemert-Pijnen JE, Nijland N, Ossebaard HC, Hendrix RM, Seydel ER (2011). Why business modeling is crucial in the development of eHealth technologies. J Med Internet Res.

[50] Mars M, Scott RE (2017). Being spontaneous: the future of Telehealth implementation?. Telemed J E Health.

[51] Institute for Health Metrics and Evaluation (IHME). Global Burden of Disease study. Botswana. Institute for Health Metrics and Evaluation (IHME). 2019. http://www.healthdata.org/botswana. Accessed 11 June 2019.

[52] Scott RE, Mars M (2013). Principles and framework for eHealth strategy development. J Med Internet Res.

[53] Novillo-Ortiz D, D’Agostino M, Becerra-Posada F (2016). Role of PAHO/WHO in eHealth capacity building in the Americas: analysis of the 2011–2015 period. Rev Panam Salud Publica.

